# Intravenous thrombolysis for acute ischemic stroke during two COVID-19 outbreaks in China: Wuhan pandemic and Beijing pandemic

**DOI:** 10.1186/s12883-023-03211-9

**Published:** 2023-05-25

**Authors:** Guangshuo Li, Shang Wang, Chuanying Wang, Yahui Hao, Yunyun Xiong, Zeyu Ding, Xingquan Zhao

**Affiliations:** 1 Department of Neurology, Beijing Tiantan Hospital, Capital Medical University, Beijing, China; 2 China National Clinical Research Center for Neurological Diseases, Fengtai District, No. 119 Nansihuanxilu, Beijing, 100070 China

**Keywords:** Stroke, Thrombolysis, Tissue plasminogen activator, COVID-19, SARS-CoV-2

## Abstract

**Introduction:**

The COVID-19 pandemic has had an impact on the emergency department (ED). Door-to-needle time (DNT) could be prolonged for intravenous thrombolysis (IVT) treatment. We aimed to investigate the impact of two COVID-19 pandemics on the workflow of IVT in our neurovascular ED.

**Method:**

We performed a retrospective analysis of patients who received IVT treatment in the neurovascular ED of Beijing Tiantan Hospital, Beijing, from January 20, 2020, to October 30, 2020, covering two COVID-19 pandemics in China. The time-based performances of IVT treatment including onset-to-arrival time, arrival-to-CT time, CT-to-needle time, door-to-needle time, and onset-to-needle time were recorded. Data on clinical characteristics and imaging information were also collected.

**Results:**

Four hundred forty patients that received IVT were enrolled in this study. The number of patients admitted to our neurovascular ED began to decrease in December 2019 and was the lowest in April 2020 (*n* = 95). Longer DNT (Wuhan pandemic: 49.00 [35.00, 64.00] min; Beijing pandemic: 55.00 [45.50, 77.00] min) interval delays were observed during the two pandemics (*p* = .016). More patients admitted during the two pandemics had an ‘unknown’ subtype (Wuhan pandemic: 21.8%; Beijing pandemic: 31.4%. *p* = .008). The percentage of the cardiac embolism subtype was higher during the Wuhan pandemic (20.0%) than during other periods. The median admission NIHSS score increased during the Wuhan pandemic and the Beijing pandemic (8.00 [4.00, 12.00], 7.00 [4.50, 14.00], respectively, *p* < .001).

**Conclusion:**

The number of patients who received IVT decreased during the Wuhan pandemic. Higher admission NIHSS scores and prolonged DNT intervals were also observed during the Wuhan pandemic and the Beijing pandemic.

## Introduction

The COVID-19 pandemic has had a great impact on global medical systems including the neurology department and other internal medicine departments [1].

Stroke ranked the 3^rd^ among the top 10 causes of disability-adjusted life-years in 2019 [2] and was a global burden disease during the COVID-19 pandemic. Intravenous thrombolysis (IVT), a highly time-sensitive treatment [3], is the most effective therapy for ischemic stroke [4], and neurovascular emergency is one of the most effective measures to perform IVT treatment as soon as possible after the onset of ischemic stroke [5]. During the COVID-19 pandemic, emergency rooms were the first barrier against the spread of COVID-19. Within emergency rooms, neurovascular emergencies also delay the treatment of IVT [6] due to the utilization of decontamination equipment and reverse- transcription PCR analysis of nasopharyngeal swabs for COVID-19 [7–9]. How to decrease the risk of exposure to COVID-19 without delaying rapid IVT treatment in neurovascular emergencies became a new issue for neurologists during the COVID-19 pandemic.

We retrospectively analyzed the data from our stroke center before, during and after the two COVID-19 pandemics in China to investigate the impact of the COVID-19 pandemic on IVT treatment to help improve the workflow of IVT treatment in neurovascular emergencies.

## Methods

### Study design

We performed a retrospective analysis of patients who received IVT treatment in the neurovascular emergency department of Beijing Tiantan Hospital, Beijing, from January 20, 2020, to October 30, 2020, covering two COVID-19 pandemics in China (one pandemic in Wuhan from January 20, 2020, and one pandemic in Beijing from June 11, 2020, based on reports from the local government). Pandemic response measures, including lockdowns, were triggered in Wuhan in January 2020, and it was the first COVID-19 pandemic in China. Xinfadi is a large market of vegetables and fruit near our stroke center at Beijing Tiantan Hospital in Beijing. Xinfadi experienced a COVID-19 pandemic in June 2020, and the emergency rooms of Beijing Tiantan Hospital, including its neurovascular emergency department, were influenced. Patients who received IVT treatment from January 2019 to October 2019 were included as a reference. Based on the periods of the two pandemics, we divided our participants into 5 groups: pre-COVID-19 group, from January 1, 2019 to October 30, 2019; Wuhan pandemic group, from January 20, 2020 to April 26, 2020; post-Wuhan-pandemic group, from April 27, 2020 to June 10, 2020; Beijing pandemic group, from June 11, 2020 to July 21, 2020; and post-Beijing-pandemic group, from July 22, 2020 to October 30, 2020.

The study was conducted in accordance with the Declaration of Helsinki. The fully deidentified data on the patients enrolled in the current study and its retrospective study design enabled this study to be conducted under a waiver of informed consent by the local institutional review board. The study was approved by the ethics committee of Beijing Tiantan Hospital (No.: KY2015-001–01). This manuscript was based on the Strengthening the Reporting of Observational Studies in Epidemiology statement to report the results of the current study.

### Participants and data collection

All patients who received IVT treatment were consecutively enrolled. The inclusion criteria were as follows: 1) diagnosed with acute ischemic stroke in our neurovascular emergency department; 2) received IVT with alteplase (0.9 mg/kg) within 4.5 h from symptoms onset; and 3) had a cranial CT/MR scan within 24 h of IVT treatment.

The demographic information among the enrolled patients included age, sex, medical history, alcohol and tobacco intake and medication history. Onset-to-arrival time (defined as the time from stroke onset to hospital arrival), arrival-to-CT time (from hospital arrival to the time of the first CT scan), CT-to-needle time (defined as time of the first CT scan to time of the IVT initiation), door-to-needle time (from the hospital arrival to the time of IVT initiation) and onset-to-needle time (from stroke onset to time of the IVT initiation) were recorded to evaluate the influence of the COVID-19 pandemic on the workflow of IVT treatment in our neurovascular emergency department. Patients confirmed to have large vessel occlusion of the anterior circulation were evaluated if they were eligible for bridging mechanical thrombectomy (MT) therapy. Stroke severity was measured by the NIHSS score [10]. The Trial of Org 10,172 in Acute Stroke Treatment (TOAST) [11] classification was also recorded. The TOAST classification denotes five subtypes of ischemic stroke: 1) large-artery atherosclerosis; 2) cardioembolism (CE); 3) small-vessel occlusion; 4) stroke of other determined etiology; and 5) stroke of undetermined etiology. The results of laboratory tests before IVT were recorded. Imaging information included the Alberta Stroke Program Early CT Score (ASPECTS) [12] before IVT treatment and the Fazekas scale score on MR scan within 24 h of IVT treatment. Symptomatic intracranial hemorrhage events were also recorded based on European cooperative acute stroke study (ECASS) II and safe implementation of thrombolysis in stroke (SITS) standards.

### Statistical analysis

Continuous variables are expressed as the mean ± SD for normally distributed data and median (interquartile range) for nonnormally distributed data. Categorical variables are expressed as numbers (percentage). Normally distributed data were compared using one-way ANOVA, and nonnormally distributed data were compared using the Kruskal–Wallis test. Categorical variables were compared using the χ2 test, as appropriate. All statistical analyses were performed with SPSS 26.0. A *P* value < 0.05 was considered statistically significant.

## Results

In the final study, 440 patients who received IVT were enrolled. In the pre-COVID-19 group, 258 patients were included as references. During the Wuhan pandemic, 55 patients were included, and 32 patients were included between the Wuhan and Beijing pandemics. During the Beijing pandemic, 35 patients were included, and 60 patients were included after the Beijing pandemic. Higher median ages were observed during the Wuhan and Beijing pandemics, but the differences were nonsignificant. (Wuhan pandemic: 65.00 [57.00, 73.00]; Beijing pandemic: 67.00 [56.00, 76.50]. *p* = 0.072). The percentages of males during the Wuhan and Beijing pandemics were 70.7% and 82.9%, respectively (shown in Tables 1 and 2).

**Table 1:** Clinical characteristics of the patients that received IVT treatment

	**Overall (*n* = 440)**	**Pre-COVID-19 (*n* = 258)**	**Wuhan pandemic (*n* = 55)**	**Post-Wuhan (*n* = 32)**	**Beijing pandemic (*n* = 35)**	**Post-Beijing (*n* = 60)**	***P* value**
**Age (years), median [IQR]**	63 [56–71]	62 [55–69]	65 [57–73]	63 [56–72]	67 [56–77]	65 [58–74]	.072
**Age ≥ 80 years, n(%)**	45 (10.2)	18 (7.0)	5 (9.1)	5 (15.6)	5 (14.3)	12 (20.0)	.043
**Male, n(%)**	310 (70.5)	179 (69.4)	39 (70.9)	22 (68.8)	29 (82.9)	41 (68.3)	.573
**Transferred, n(%)**	49 (11.1)	32 (12.4)	5 (9.1)	5 (15.6)	2 (5.7)	5 (8.3)	.559
**Bridging MT, n(%)**	43 (9.8)	22 (8.5)	5 (9.1)	0 (0.0)	9 (25.7)	7 (11.7)	.006
**Onset-to-arrival time (min), median [IQR]**	114.50 [73.00–165.00]	120.00 [70.75–169.25]	100.00 [70.50–149.50]	117.50 [73.25–138.75]	110.00 [75.50–185.00]	120.50 [77.25–164.75]	.556
**Arrival-to-CT time (min), median [IQR]**	16.00 [10.00–24.00]	15.00 [8.00–21.00]	17.00 [11.00–22.75]	16.00 [10.00–30.00]	25.00 [16.00–33.00]	20.00 [13.75–28.00]	< .001
**CT-to-tPA (min), median [IQR]**	29.00 [21.00–51.25]	29.00 [20.25–52.00]	31.00 [21.50–52.00]	35.00 [23.50–106.25]	30.00 [23.50–45.00]	26.50 [19.75–41.00]	.372
**DNT time (min), median [IQR]**	45.00 [35.00–61.00]	43.00 [33.00–58.00]	49.00 [35.00–64.00]	48.50 [39.75–70.50]	55.00 [45.50–70.00]	47.00 [38.00–60.50]	.016
**DNT ≤ 60 min, n(%)**	325 (73.9)	200 (77.5)	38 (69.1)	20 (62.5)	22 (62.9)	45 (75.0)	.146
**Onset-to-tPA (min), median [IQR]**	167.50 [126.75-220.50]	166.50 [125.25–218.00]	155.00 [123.00–204.50]	170.00 [132.25, 219.25]	169.00 [132.50–244.00]	174.50 [126.00–227.00]	.597
**Pre-mRS score, n(%)**							.155
**0**	291 (76.6)	179 (75.2)	39 (88.6)	20 (74.1)	19 (79.2)	34 (72.3)	
**1**	44 (11.6)	29 (12.2)	0 (0.0)	3 (11.1)	4 (16.7)	8 (17.0)	
**2**	21 (5.5)	14 (5.9)	2 (4.5)	1 (3.7)	0 (0.0)	4 (8.5)	
**3**	14 (3.7)	7 (2.9)	3 (6.8)	2 (7.4)	1 (4.2)	1 (2.1)	
**4**	9 (2.4)	8 (3.4)	0 (0.0)	1 (3.7)	0 (0.0)	0 (0.0)	
**5**	1 (0.3)	1 (0.4)	0 (0.0)	0 (0.0)	0 (0.0)	0 (0.0)	
**Admission NIHSS score, median [IQR]**	5 [3-11]	5 [3-9]	8 [4-12]	6 [4-10]	7 [5-14]	9 [4-12]	< .001
**NIHSS score ≤ 3, n (%)**	126 (28.6)	91 (35.3)	9 (16.4)	8 (25.0)	6 (17.1)	12 (20.0)	.006
**NIHSS score ≤ 5, n (%)**	227 (51.6)	148 (57.4)	25 (45.5)	16 (50.0)	15 (42.9)	23 (38.3)	.048
**NIHSS score after tPA injection, median [IQR]**	3 [1-7]	3 [1–6]	5 [2–10]	3 [1–7]	5 [2–14]	3 [1–9]	.013
**NIHSS score at 2 h after tPA injection, median [IQR]**	4 [1–7]	2 [1–6]	3 [2–5]	4 [1–8]	4 [3, 13]	4 [1–11]	.411
**NIHSS score at 24 h after tPA injection, median [IQR]**	3 [1–7]	2 [1–5]	3 [1–10]	4 [0–6]	6 [3–12]	3 [1–9]	.275
**NIHSS reduction at 24 h, (median [IQR])**	2 [0–4]	2 [0–4]	3 [0–4]	2 [0–3]	2 [-1–6]	2 [0–5]	.915
**Hypertension, n(%)**	225 (51.1)	138 (53.5)	29 (52.7)	18 (56.2)	13 (37.1)	27 (45.0)	.330
**Atrial fibrillation, n(%)**	38 (8.6)	25 (9.7)	5 (9.1)	1 (3.1)	1 (2.9)	6 (10.0)	.398
**Diabetes, n(%)**	101 (23.0)	62 (24.0)	10 (18.2)	11 (34.4)	3 (8.6)	15 (25.0)	.113
**Hyperlipidemia, n(%)**	48 (10.9)	34 (13.2)	4 (7.3)	4 (12.5)	3 (8.6)	3 (5.0)	.281
**Prior stroke, n(%)**	85 (19.3)	57 (22.1)	8 (14.5)	5 (15.6)	5 (14.3)	10 (16.7)	.512
**History of antiplatelet therapy, n(%)**	60 (13.6)	30 (11.6)	6 (10.9)	6 (18.8)	6 (17.1)	12 (20.0)	.389
**History of statin, n(%)**	47 (10.7)	26 (10.1)	5 (9.1)	6 (18.8)	2 (5.7)	8 (13.3)	.468
**Smoking, n(%)**	215 (48.9)	127 (49.2)	27 (49.1)	18 (56.2)	14 (40.0)	29 (48.3)	.769
**Drinking, n(%)**	179 (40.7)	108 (41.9)	22 (40.0)	16 (50.0)	10 (28.6)	23 (38.3)	.467
**TOAST, n(%)**							.008
**Large-artery atherosclerosis**	293 (66.6)	187 (72.5)	29 (52.7)	21 (65.6)	21 (60.0)	35 (58.3)	
**Cardioembolism**	58 (13.2)	34 (13.2)	11 (20.0)	4 (12.5)	2 (5.7)	7 (11.7)	
**Small-vessel occlusion**	14 (3.2)	10 (3.9)	0 (0.0)	1 (3.1)	1 (2.9)	2 (3.3)	
**Stroke of other determined etiology**	9 (2.0)	4 (1.6)	3 (5.5)	1 (3.1)	0 (0.0)	1 (1.7)	
**Stroke of undetermined etiology**	66 (15.0)	23 (8.9)	12 (21.8)	5 (15.6)	11 (31.4)	15 (25.0)	
**Admission SBP (mmHg), median [IQR]**	150 [136–166]	151 [137–166]	151 [130–160]	156 [141–166]	145 [130–165]	145 [135–170]	.804
**Admission DBP (mmHg), median [IQR]**	88 [78–96]	88 [79–96]	80 [74–93]	92 [89–98]	86 [79–95]	83 [75–94]	.294

*MT* mechanical thrombectomy, *tPA* tissue plasminogen activator, *DNT* door-to-needle, *NIHSS* national institutes of health stroke scale, *TOAST* Trial of Org 10,172 in Acute Stroke Treatment, *SBP* systolic blood pressure, *DBP* diastolic blood pressure

**Table 2:** Laboratory and imaging information of the patients that received IVT treatment

	**Overall (*n* = 440)**	**Pre-COVID-19 (*n* = 258)**	**Wuhan pandemic (*n* = 55)**	**Post-Wuhan (*n* = 32)**	**Beijing pandemic (*n* = 35)**	**Post-Beijing (*n* = 60)**	***P* value**
Serum glucose level (mmol/L), median [IQR]	6.89 [5.89–8.75]	6.84 [5.89–8.62]	7.19 [5.97–9.29]	6.84 [5.57–8.01]	6.55 [5.92–8.09]	7.50 [6.01–9.30]	.616
HbA1c (%), median [IQR]	6.00 [5.70–6.82]	6.00 [5.70–6.60]	6.00 [5.65–7.10]	6.25 [5.62–7.20]	5.95 [5.80–6.30]	6.20 [5.75–6.90]	.75
LDL level (mmol/L), mean (SD)	2.56 ± 0.84	2.68 ± 0.81	2.79 ± 0.95	2.44 ± 0.85	2.27 ± 0.87	2.19 ± 0.62	.001
Cholesterol level (mmol/L), mean (SD)	4.18 ± 0.97	4.30 ± 0.92	4.49 ± 1.07	4.09 ± 1.04	3.76 ± 0.93	3.75 ± 0.81	.001
ASPECTS score, median [IQR]	8 [7–9]	9 [8-9]	10 [10-10]	9 [8–10]	8 [7–9]	8 [6–9]	.279
MR DWI positive, n(%)	297 (86.6)	166 (83.8)	38 (86.4)	24 (92.3)	25 (89.3)	44 (93.6)	.323
Fazekas scale, median [IQR]	1 [1-2]	1 [1-1]	1 [1-2]	1 [1-2]	2 [1-2]	1 [1-2]	.001
Cranial vessel stenosis location, n(%)							.110
ACA	5 (3.2)	3 (3.5)	0 (0.0)	0 (0.0)	0 (0.0)	2 (7.7)	
MCA	77 (50.0)	43 (50.0)	10 (58.8)	4 (12.5)	7 (53.8)	13 (50.0)	
PCA	15 (9.7)	10 (11.6)	3 (17.6)	2 (16.7)	0 (0.0)	0 (0.0)	
ICA	38 (24.7)	22 (25.6)	2 (11.8)	5 (41.7)	4 (30.8)	5 (19.2)	
VA	3 (1.9)	1 (1.2)	0 (0.0)	1 (8.3)	1 (7.7)	0 (0.0)	
BA	16 (10.4)	7 (8.1)	2 (11.8)	0 (0.0)	1 (7.7)	6 (23.1)	
Stenosis degree, n(%)							.548
≤ 70%	302 (68.8)	176 (68.2)	38 (69.1)	20 (62.5)	23 (65.7)	45 (75.0)	
70–99%	50 (11.4)	33 (12.8)	7 (12.7)	5 (15.6)	2 (5.7)	3 (5.0)	
Occlusion	88 (20.0)	49 (19.0)	10 (18.2)	7 (21.9)	10 (28.6)	12 (20.0)	
sICH-ECASS II, n(%)	17 (3.9)	4 (1.6)	5 (9.1)	0 (0.0)	1 (2.9)	7 (11.7)	.002
sICH-SITS, n(%)	10 (2.3)	2 (0.8)	4 (7.3)	0 (0.0)	0 (0.0)	4 (6.7)	.001

*LDL* low density lipoprotein, *ASPECTS* alberta stroke program early CT score, *DWI* diffusion weighted imaging, *ACA* anterior cerebral artery, *MCA* middle cerebral artery, *PCA* posterior cerebral artery, *ICA* internal carotid artery, *VA* vertebral artery, *BA* basal artery, *sICH* symptomatic intracerebral hemorrhage, *ECASS* European cooperative acute stroke study, *SITS* safe implementation of thrombolysis in stroke

**Fig. 1:**
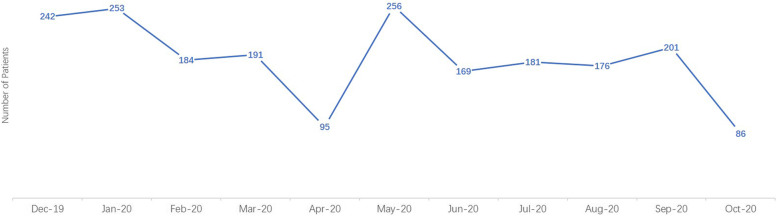
Numbers of patients admitted in our neurovascular emergency from December, 2019 to October, 2020

**Fig. 2:**
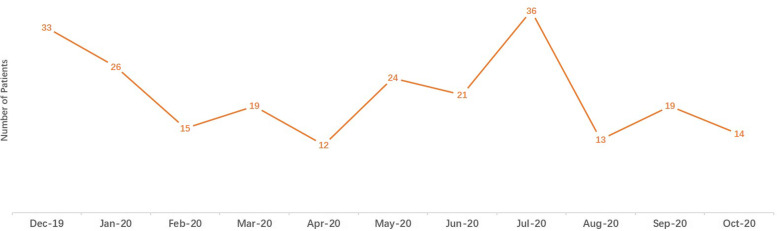
Numbers of patients that received IVT in our neurovascular emergency from December, 2019 to October, 2020

**Fig. 3:**
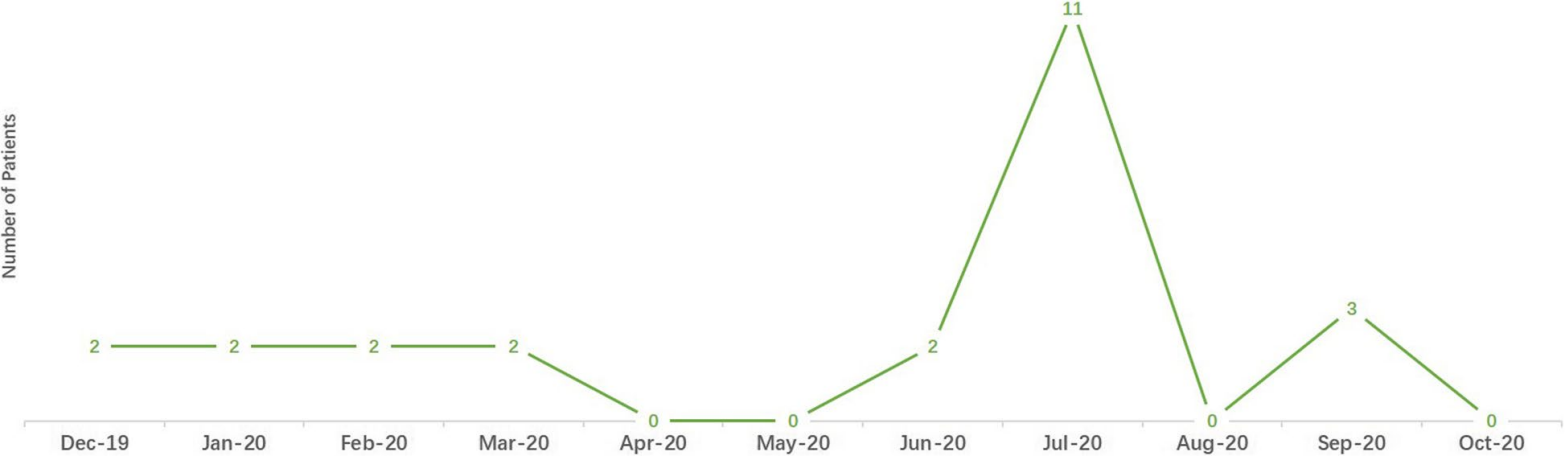
Numbers of patients that received bridging MT in our neurovascular emergency from December, 2019 to October, 2020

The number of patients admitted to our neurovascular emergency department began to decrease in December 2019 and was the lowest in April 2020 (*n* = 95) (shown in Fig. 1). In June 2020, our neurovascular emergency department had the most patients who received IVT (*n* = 36) and IVT bridging MT (*n* = 11) (shown in Figs. 2 and 3). The percentage of patients who underwent IVT bridging MT was higher during the two pandemics than during other periods (Wuhan pandemic: 9.1%; Beijing pandemic: 25.7%).

Longer DNT interval delays were observed during the two pandemics (Wuhan pandemic: 49.00 [35.00, 64.00] min; Beijing pandemic: 55.00 [45.50, 70.00] min. *p* < 0.016). However, the percentage of DNT time ≤ 60 min did not decrease significantly during the two pandemics (*p* = 0.146). Compared with the CT-to-tPA time, the arrival-to-CT time was prolonged during the two pandemics (Wuhan pandemic: 17.00 [11.00, 22.75] min; Beijing pandemic: 25.00 [16.00, 33.00] min. *p* < 0.001).

For the TOAST subtype of ischemic stroke, more patients admitted during the two pandemics had stroke of undetermined etiology (Wuhan pandemic: 21.8%; Beijing pandemic: 31.4%. *p* = 0.008). The percentage of the CE subtype during the Wuhan pandemic was higher than that during the other periods (20.0%). The median admission NIHSS score during the Wuhan pandemic increased compared with that during the pre-COVID-19 period (8.00 [4.00, 12.00] vs. 5.00 [3.00, 9.00]). The Beijing pandemic had a higher median admission NIHSS score than the Wuhan and Beijing pandemics (7.00 [4.50, 14.00] vs. 5.50 [3.75, 10.00], respectively, *p* < 0.001). While this difference remained after the intravenous tPA drip finished (*p* = 0.013), the NIHSS score was similar at 2 h (*p* = 0.411) and 24 h after IVT (*p* = 0.275). Lower percentages of minor stroke (NIHSS score ≤ 3) were observed during the two pandemics (Wuhan pandemic: 16.4%; Beijing pandemic: 17.1%. *p* = 0.006). Patients who received IVT treatment during the Wuhan pandemic had the highest percentage of sICH of the SITS classification, (7.3%) and patients who received IVT treatment after the Beijing pandemic had the highest percentage of sICH of the ECASS II classification (11.7%).

## Discussion

Our study showed a decrease in the number of patients admitted to our neurovascular emergency department since January 2020, when the Wuhan pandemic broke out. The number of patients who received IVT also showed a clear drop during the Wuhan pandemic. Prolonged DNT interval times and arrival-to-CT times were observed during the two pandemics. Patients who received IVT during the two pandemics showed more severe neurological impairment, manifesting as a higher median NIHSS score.

The lower number of patients admitted to our neurovascular emergency department could be due to the lockdown of the city and the fear of COVID-19 infection to avert going to the hospital if the symptoms of ischemic stroke were not severe. A retrospective analysis from four stroke centers in Wuhan showed a 50% reduction in patients admitted to stroke emergency departments during the Wuhan pandemic [13]. A cohort study from the Thrombolysis in Ischemic Stroke Patients (TRISP) registry that included 540 patients from 20 stroke centers in Switzerland demonstrated a 7% decrease in the number of patients who underwent IVT/MT during COVID-19 [14]. A French study found that the Alsace region had a 40.9% lower number of patients who received IVT during the COVID-19 pandemic [15]. In May 2020, the number of patients admitted to our neurovascular emergency department returned to the level observed during the pre-COVID-19 period, and this number began to decrease again in June 2020, when the Beijing pandemic broke out. A similar decline was also observed in a large-scale cohort study that included over 10,000 patients from 56 countries [16]. Our stroke emergency department had more stroke patients who received IVT during July 2020 when the Beijing pandemic broke out. The Beijing pandemic originated from Xinfadi, a large market near our hospital. During the Beijing pandemic, many neurologists were transferred to the fever clinic of their hospitals, and the ability to receive stroke patients, including performing IVT, was reduced in these hospitals. Our stroke emergency department, as the unique senior stroke center around Xinfadi, received more stroke patients. Based on our data, a nearby COVID-19 pandemic may have a different impact on a comprehensive stroke center compared to a pandemic far from the stroke center; a nearby COVID-19 pandemic may increase the burden of stroke patients for a stroke center. However, this speculation was derived from our single-center analysis and may be less generalizable until similar studies are conducted.

**Fig. 4:**
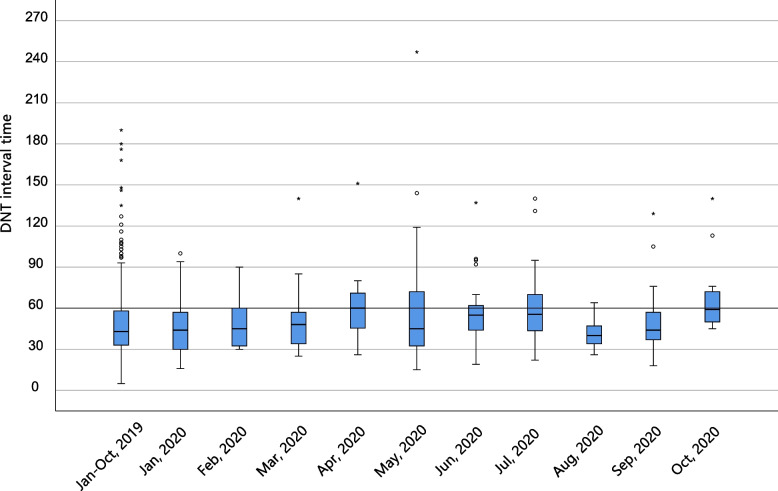
DNT interval time of IVT in our neurovascular emergency from December, 2019 to October, 2020

**Fig. 5:**
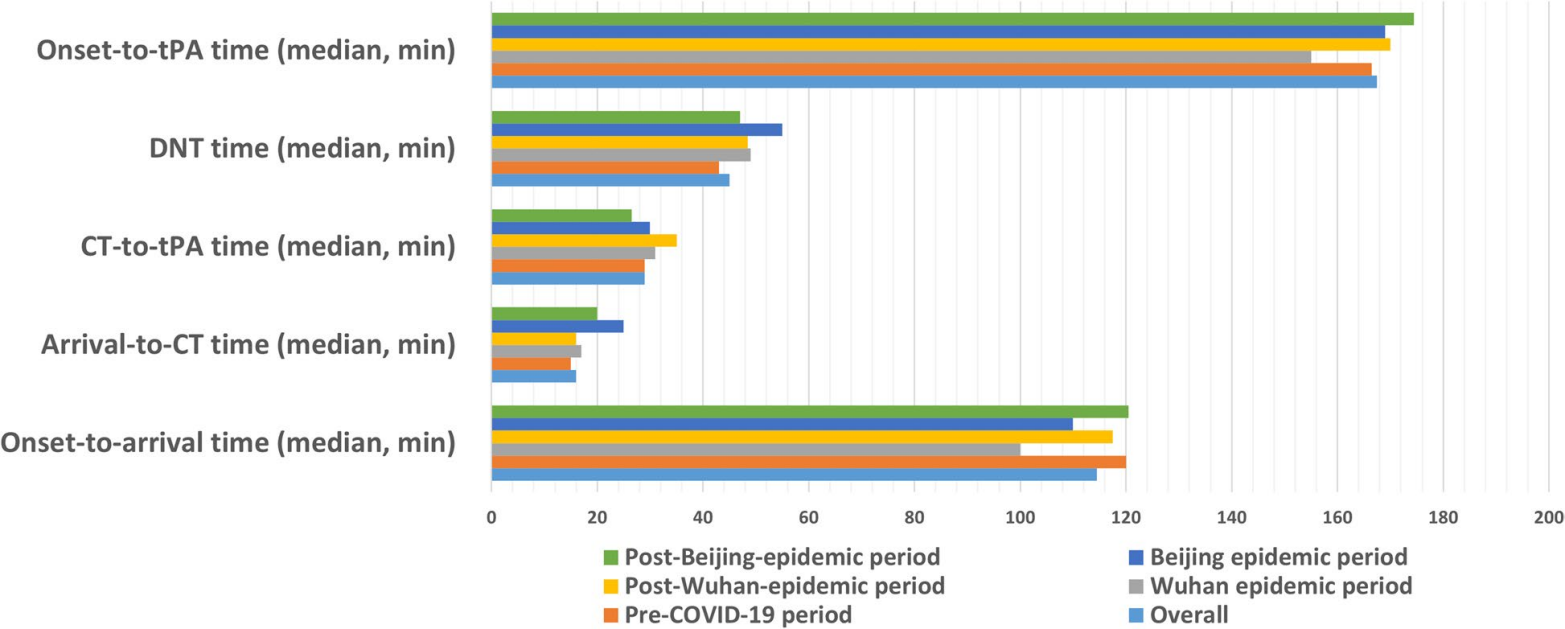
The time-based performances of IVT treatment in our neurovascular emergency

The DNT interval time, particularly the arrival-to-CT time, was prolonged during the two pandemics. DNT time began to increase from January, 2019 to April, 2020. In May, 2020, when the Wuhan pandemic ceased, the DNT time decreased but increased again from June, 2020 when the Beijing pandemic broke out (shown in Fig. 4). A longer DNT delay of approximately 5 min was also presented in another comprehensive tertiary stroke center due to the COVID-19 pandemic [17]. This might be due to the additional measures against the spread of COVID-19. All the patients and their relatives were required to fill out an epidemiological questionnaire before entering the emergency room. Fewer neurologists in neurovascular emergency departments and more complicated prehospital assessments contributed to the delay in DNT time and arrival-to-CT time, including the collection of nasopharyngeal swabs for COVID-19 PCR analysis, sterilization and body temperature measurements. The retrospective analysis from four Wuhan hospitals showed that the DNT time was doubled during the Wuhan pandemic [13], a much more severe delay compared with that of our stroke emergency because these four Wuhan hospitals experienced the COVID-19 pandemic earlier than our stroke center without sufficient preparation. A pooled analysis that enrolled 1464 stroke patients during COVID-19 from 14 comprehensive stroke centers in the US during the COVID-19 pandemic showed lower odds of a DNT interval < 60 min, mainly due to a prolonged delay of the imaging-to-needle interval [6]. Extra delay may be due to decontamination of critical health care resources and donning of personal protective equipment in the US. Our study also showed that during the period between the Wuhan pandemic and the Beijing pandemic, the DNT time was still longer compared with that of the pre-COVID-19 period, indicating that the impact of the COVID-19 pandemic could persist because of the maintaining measures, including nasopharyngeal swabs test, measure of sterilization, taking swab testing, sterilization measures, body temperature measurements and filling out of an epidemiological questionnaire. After the Beijing pandemic, the DNT time decreased compared with that during the period between the Wuhan pandemic and the Beijing pandemic but was still longer compared with that during the pre-COVID-19 period. As the Chinese government claimed ‘Normalization of the COVID-19 pandemic’, public hospitals in China would maintain these measures for a long time to prevent the spread of COVID-19 for a long time in the future, and the DNT time may be prolonged and hard to return to the level as that during the pre-COVID-19 period. Onset-to-arrival time decreased during the two pandemics, which could be due to less traffic jam in Beijing because local residents tended to stay home for fear of COVID-19 (shown in Fig. 5). Another observational study showed a paradoxically shortened DNT time compared with our study [18]. This was attributed to the decreased number of stroke patients admitted to that stroke center, while the decrease was not observed in our stroke center.

Higher baseline NIHSS scores were observed during the two pandemics in our neurovascular emergency department, indicating an increased severity of stroke patients who received IVT therapy. On one hand, COVID-19 might deteriorate neurological impairment and worsen clinical outcomes [19, 20]. However, no patients in our neurovascular emergency department were confirmed to be COVID-19 positive. On the other hand, during the two pandemics, patients tended to resist going to the hospital, fearing in-hospital COVID-19 [21]. Patients who went to the hospital had more severe and fatal symptoms [6]. As a result, in our study, the median baseline NIHSS score was higher during the two pandemics. Four Wuhan hospitals also admitted a nonsignificantly higher baseline NIHSS score during 2020 than during 2019 [19]. Moreover, the percentage of patients who received bridging thrombectomy was higher during the two pandemics in our center, implying that more strokes were due to intracranial large vessel occlusion and that patients had severe stroke symptoms.

During the Wuhan pandemic, our neurovascular emergency department received more patients with the CE subtype (20.00%). However, during the Beijing pandemic, the percentage of CE subtypes was the lowest (5.71%). Many patients receiving IVT rejected admission to neurology inpatient wards to complete further examinations on the TOAST subtype during the Beijing pandemic. Some patients with the CE subtype might be classified into the unknown subtype (30%). During the Wuhan pandemic, four stroke centers from Wuhan also admitted more patients with the CE subtype and an elevated percentage of stroke of undetermined etiology, which is consistent with our study [19]. A large, multicenter cohort from 14 comprehensive stroke centers in the US also demonstrated an increased percentage of the CE subtype with more severe neurological impairment [6]. The percentage of sICH was different among the different groups. Even if the differences were statistically significant, we were not able to introduce conclusions because the number of patients with sICH events was too low.

As the global pandemic of COVID-19 shows no tendency to cease, stroke centers are faced with its impact on neurovascular emergencies, including prolonged DNT time and a shortage of stroke team members. Studies on the workflow of neurovascular emergencies are warranted to minimize the DNT delay and door-to-puncture time to achieve rapid recanalization and more favorable functional outcomes [22]. Most of the studies only investigated the impact of one COVID-19 pandemic on stroke emergencies. Our study provides a retrospective analysis of patients who received IVT from a senior stroke center that underwent two COVID-19 pandemics in China. The two COVID-19 pandemics consisted of the Wuhan pandemic, which was far from our stroke center, and the Xinfadi pandemic, which was close to our center. The comparison of the two different pandemics showed unique results that have rarely been observed in previous studies.

Several limitations have to be admitted in our study. The retrospective and single-center study design may introduce selection bias. However, as the Chinese government transferred many doctors to fever clinics during the COVID-19 pandemics, stroke emergency and neurology inpatient wards lacked doctors to conduct a prospective cohort or registry study that included hospitals from different regions. Second, the sample size of our study may be small. However, our single-center, retrospective study did not depend on a large study sample, and our study provided a larger sample than another retrospective study from China [13]. Third, our study lacked the follow-up information of the included patients. According to the claim of ‘normalization’ of the COVID-19 pandemic proposed by the Chinese government, our neurovascular emergency department is lacking neurologists to perform follow-up. However, as short-term outcomes, our analysis showed that the NIHSS scores at 2 h and 24 h after IVT treatment were similar between the pandemic and nonpandemic groups, suggesting that the long-term outcomes may not be different between the two groups.

## Conclusion

Our study found that the number of patients who received IVT decreased during the Wuhan pandemic. Higher admission NIHSS scores and prolonged DNT intervals were also observed during the Wuhan pandemic and the Beijing pandemic.

## Data Availability

The datasets used and/or analysed during the current study available from the corresponding author on reasonable request.
